# The Book World of Medicine and Science

**Published:** 1911-07-22

**Authors:** 


### THE BOOK WORLD OF MEDICINE AND SCIENCE.

Vicious Circles in Disease. By Jamieson B. Hurry, M.D., M.A. Illustrated. (London: J. and A. Churchill. 1911. Demy 8vo. Pp. 186. Price 6s. net.)

The application of the principle of the vicious circle to a large number of morbid conditions is by no means difficult, and the array of vicious circles brought together in this monograph does not necessarily justify the elevation of their study to the importance which Dr. Hurry endeavours to claim for them. In a very elegantly bound and printed volume we are treated to an erudite discussion of innumerable diseases and symptoms, all of which have a vicious circle attached to them by the author. We venture to think that the principle might even have been extended still further and applied with a little ingenuity to such a complaint as housemaid's knee. The author must have derived great pleasure in compiling such an agreeable book as this is, and his friends will, we are sure, find its perusal a great relief from the ordinary prosaic text-book; while the large number of quotations in Greek and Latin will help them to brush up their classics. Much in this monograph is merely the exposition of the obvious; yet it is useful to have collected together these complex correlations, which, as the author hopes, may "unravel many a Gordian knot in obscure disease and lead to increased accuracy of diagnosis." The contents show a breadth of view and a keenness of insight born of a long and careful study of diseased humanity only possible in a large general practice. We cordially hope that the work will obtain a wide distribution if only for its excellent philosophy and classical character.

The Cancer Problem: A Statistical Study. By C. E. Green, F.R.S.E. (Edinburgh: William Green and Sons. 1911.)

This book represents an earnest endeavour to apply the deductive method to the problem of cancer causation. The matter shows evidence of much care and pains, and is in itself very interesting reading; although the subtitle leads one to expect a deluge of statistics, these do not form the mainstay of the treatise, which deals with parasitic fungi, occupational and local incidence, and the importance of soot and sulpho-acids. The author wishes to present a case for cancer being due to a parasite belonging to the Mycetozoa and akin to the *Plasmodiophoria brassicæ*, the growth of which is promoted by conditions allowing of access of $SO_2$ and other sulpho-acids, while it is adversely affected by the presence of calcium salts. Statistics relating to the incidence of cancer among the workers in certain trades and the dwellers in smoky atmospheres or in localities situated in hollows are given in some detail and, according to the author, these point to the existence of a relation between the above-mentioned factors and the cancer death-rate under the conditions of life pertaining to such communities.

Golden Rules of Ophthalmic Practice. By Gustavus Hartridge, F.R.C.S. Fifth edition. 1911. (Bristol: John Wright and Sons. Price 1s.)

With the object of accentuating the essential points in eye disease and to form an elementary framework which may be constantly added to, the author has written a useful volume in the popular "Golden Rule Series." A fifth edition having been called for, it is obvious that this member of the Series has been appreciated, and Mr. Hartridge may be congratulated in having successfully gathered together in so small a compass so many practical points and brief but serviceable summaries.

The Bacillus of Long Life: A Manual on the Preparation of Soured Milk. By Loudon M. Douglas, F.R.S.E. With many illustrations. (London: T. C. & E. C. Jack. 1911. Price 5s.)

Around the subject of sour milk and its therapeutic value much controversy has raged and remarkable differences of opinion have been evoked. It can hardly be said that the nihilistic assertions of the scoffers have as yet predominated, and there is still evinced a large amount of interest in the subject. It is hardly to be expected that desultory trials can compare in effect with the results obtained in those countries where a life-long indulgence in a diet of fermented milk is a common occurrence. Mr. L. M. Douglas has opportunely produced an instructive and comprehensive compilation of the literature on the subject. The title he has chosen leaves no doubt as to his convictions regarding the efficacy of the working of the bacilli concerned in lactic fermentation towards the attainment of long life. While one does not look for a scientific treatise in a book with such a title, it will be found that there is presented a large amount of bacteriological and chemical matter. The author points out the inefficiency, not to say danger of some of the commercial preparations, and goes fully into the bacteriology of the large number of organisms associated with the varying methods employed in different countries. He concludes that a culture prepared by the use of organisms of the type *Streptococcus lacticus* combined with the *Bacillus Bulgaricus* is the most satisfactory and possesses a more agreeable flavour than those prepared from a pure culture of *Bulgaricus* alone. The book is provided with an unusually large number of illustrations. Plates with micro-photographs of bacteria follow one another with almost bewildering profuseness. There are also numerous excellent photographs of scenes of peasant life in the Balkans and other views, which are only remotely relevant to the subject. The historical portion of the book makes very interesting reading, touching as it does the customs of many lands, and should help considerably the author's case. Included in the manual are clear and concise directions concerning the preparation of soured milk, both in the household and on a larger scale in the dairy; together with descriptions and illustrations of some of the apparatus specially devised and readily obtainable for that purpose. It is reassuring to know that a manual of this character is available in which the whole question of sour milk is entered into in a scientific spirit.

Regimen Sanitatis: A Gaelic Medical Manuscript of the Early Sixteenth Century. Edited by H. Cameron Gillies, M.D. (Printed for the Author by R. Maclehose and Co., Glasgow. 1911. Pp. 139.)

The tract which is here reproduced, first in facsimile, then in transliteration, next translated into English, and finally extensively annotated, is an original Gaelic manuscript from the British Museum. It belonged to John MacBeath, one of a family of hereditary physicians to the Lords of the Isles and to the Kings of Scotland for several centuries. Of the industry of the editor there can be no doubt; as to his Gaelic scholarship the present critic does not feel competent to judge, but at least in regard to mediæval medicine his erudition is apparent. In the pathology and ætiology of present-day diseases it is not quite so easy to follow him; thus, in a note he upholds stoutly a theory that appendicitis is caused solely by restraining the natural inclination to empty the colon when certain sensations arise. In the preface an egotistical tone is struck which would certainly have been much better avoided. The book is a very valuable contribution to the history of medicine.

